# The role of physical activity in the association between disability and mortality among US older adults: a nationwide prospective cohort study

**DOI:** 10.1007/s11357-024-01072-9

**Published:** 2024-01-22

**Authors:** Rocio Izquierdo-Gomez, David Martínez-Gómez, Nora Shields, María del Rosario Ortola-Vidal, Fernando Rodríguez-Artalejo, Verónica Cabanas-Sánchez

**Affiliations:** 1 GALENO Research Group, Department of Physical Education, Faculty of Education Sciences, University of Cádiz, Cádiz, Spain; 2 Biomedical Research and Innovation Institute of Cádiz (INiBICA), Research Unit, Cádiz, Spain; 3 Universidad de Cádiz, Avda. República Saharahui, S/N. Campus de Puerto Real, 11519 Cádiz, Puerto Real Spain; 4 Department of Preventive Medicine and Public Health, School of Medicine, Universidad Autónoma de Madrid, Madrid, Spain; 5 CIBERESP (CIBER of Epidemiology and Public Health), Madrid, Spain; 6 IMDEA-Food Institute, CEI UAM+CSIC, Madrid, Spain; 7 Olga Tennison Autism Research Centre, School of Psychology and Public Health, La Trobe University, Victoria, 3086 Australia

**Keywords:** Disability, Aging, Survival, Physical activity

## Abstract

To examine whether physical activity can mitigate the mortality risk associated with disability in activities of daily living and instrumental activities in daily living among older adults. This analysis comprised 177,360 US participants (104,556 women), aged ≥ 60 years, with valid data from the 1997 through 2018 waves of the US National Health Interview Survey. Participants reported the frequency and duration of leisure-time PA, and their disabilities in activities of daily living and instrumental activities in daily living. Mortality data were obtained from the National Death Index. Over a mean (SD) follow-up of 8.02 (5.43) years, 66,694 deaths occurred from all-cause, 22,673 from cardiovascular disease, and 13,845 from cancer. Among people with disability in activities in daily living, those reaching physical activity recommendations had 25%, 24% and 33% lower risk of all-cause mortality, cardiovascular diseases, and cancer death, respectively, compared with those who do not meet physical activity recommendations. Values were 23%, 22% and 24% for those with disability in instrumental activities in daily living. Risk reductions associated with reaching the recommended physical activity ranged 16% to 29% for people without disability. Combining disability type and compliance with physical activity, individuals with disability in activities of daily living or instrumental activities in daily living who meet the recommended physical activity had moderately higher mortality than those without disability who did not achieve the recommended physical activity. Compliance with physical activity recommendations can partially mitigate excess mortality resulting from disability in activities in daily living or instrumental activities in daily living in older adults.

**Supplementary Information:**

The online version contains supplementary material available at 10.1007/s11357-024-01072-9.

## Introduction

Disability in older people is a major social and public health challenge worldwide, particularly in the context of aging population [1, 2]; since the prevalence of disability increases with age, late-life disability is expected to further increase the burden on health and social care system in the next decades [3]. Disability is a complex concept, but in older people it usually refers to limitations in the individual capacity to perform activities of daily living (ADLs) or instrumental activities of daily living (IADLs), due to physical, cognitive or mental health conditions [4]. Disability in older adults has been associated with poor health outcomes and increased mortality [5–7]. For example, Yang et al. [8] found that disability in ADLs had a strong impact on all-cause mortality in a Chinese longitudinal older population-based study. Another study reported that the presence of disability in ADLs or IADLs was associated with an increase in all-cause mortality risk in elderly US population aged 60 to 84 years [6]. Martinez-Gomez et al. [9] reported a higher mortality risk in all-cause and CVD in a representative sample of older Spanish individuals with disability in global daily activities and IADLs compared with those without disability conditions. This underscores the need to improve the health status of older adults with disabilities, as well as develop strategies to avert the functional decline or mortality resulting from their disabling conditions.

Physical activity (PA) plays an important role in maintaining or improving functional status and reducing mortality in older adults [10–13]. Previous evidence suggests that meeting PA recommendations (i.e., 150 min of moderate-to-vigorous PA per week [10]) effectively reduces all-cause mortality in the general older population; [10, 14, 15] also, PA partially mitigates the increased risk of mortality in older adults with physical disabilities [9]. However, the magnitude of the attenuation of mortality resulting from PA in older people with disability in ADLs and IADLs is uncertain. [9, 11] Accordingly, using data from a large population-based US cohort, this study examined: (i) the risk of all-cause, cardiovascular disease (CVD) and cancer mortality related to having disability in ADLs and IADLs, (ii) the association between PA and all-cause, CVD and cancer mortality among people with and without disability in ADLs and IADLs, and (iii) the combined association of disability (i.e., ADLs, IADLs) and meeting PA recommendations with all-cause, CVD and cancer mortality in older adults.

## Methods

### Study design and participants

This analysis included combined data from the 1997 to 2018 waves of the US National Health Examination Survey (NHIS), a cross-sectional study annually conducted by the Centers for Disease Control and Prevention (CDC) and the National Center for Health Statistics (NCHS). The NHIS uses a multi-stage probability sampling designed to collect nationally representative estimates of the US civilian, noninstitutionalized population in the US. Data were collected via in-person household computer-assisted interviews were conducted, with telephone follow-up when the interview cannot be completed. The Complete NHIS methods and procedures are publicly available on NHIS website (https://www.cdc.gov/nchs/nhis/).

Our study population was restricted to 1997–2018 NHIS adults with valid records and aged ≥ 60 years. A total of 183,604 participants with available information on mortality was initially included. After excluding 6,244 participants without complete data on disability (*n* = 785) or PA (*n* = 5,459), the analytical sample of this study comprised 177,360 US older adults (Supplementary Fig. 1). All participants provided informed consent prior to being included in the survey, and all NHIS content and procedures were approved by the NCHS Research Ethics Review Board. No additional institutional approval was required to conduct secondary analyses using NHIS public-use data.

## Measures

### Disability

Participants were asked if they needed the help of another person for six personal care activities (bathing or showering, dressing, eating, using or getting to the toilet, getting around inside the home, and getting in/out of bed or chairs) because of a physical, mental or emotional problem [16]. A person was considered to have disability in ADLs if they needed help to perform one or more essential activities for personal self-care.

Participants were also asked to indicate, in one question, difficulties in handling routine needs, such as everyday household chores, doing necessary business, shopping, or getting around for other purposes [16]. A person was considered to have disability in IADLs if they needed assistance to perform routine activities.

#### Leisure-time physical activity

Light-moderate and vigorous leisure-time PA was recorded in terms of frequency and duration [16]. Subsequently, time per week (min/week) in light-moderate (MPA) and vigorous PA (VPA) was calculated, and PA was estimated as MPA + 2*VPA [17]. In order to correct over-reporting and minimize the influence of outliers on results, PA was truncated to 1,680 min/week [18]. PA was also classified according to the 2020 WHO PA guidelines [10] into: (i) not meeting WHO recommendations (PA < 150 min/week), and (ii) meeting WHO recommendations (PA ≥ 150 min/week).

### Mortality ascertainment

The leading underlying cause of death was obtained by the linkage of the participant data with the National Death Index (NDI) mortality data, based on probabilistic matches [19]. NCHS classifies the underlying cause of death into 10 categories, using the 10th revision of the International Statistical Classification of Diseases, Injuries, and Causes of Death (ICD-10). All-cause mortality, as well as CVD (ICD-10 codes: I00-I99) and cancer mortality (ICD-10 codes: C00-C97) were considered. Follow-up period (in months) was calculated from the interview date to the date of death for deceased participants or to censoring date (31 December 2019) for the rest of participants.

### Covariates

Selected covariables, including sociodemographic, lifestyle, and health-related factors that have been relevant on the previous scientific literature and availability in the NHIS dataset [9, 20]. Sociodemographic covariates included sex (men; women), age (years), ethnicity (non-Hispanic White; non-Hispanic Black; Hispanic; and other race), educational attainment (less than high school; high school grade or equivalent; and more than high school), and relationship status (married or living with a partner; divorced, separated, or widowed; and never married). Lifestyle covariates were smoking status (never; former; current) and alcohol drinking (never; former; current). As health-related factors, body mass index (BMI) was calculated as self-reported weight (in kg) divided by squared self-reported height (in cm) and classified as underweight or normal-weight (< 25 kg/m^2^), overweight (25–29.9 kg/m^2^), and obesity (≥ 30 kg/m^2^). Participants also reported if they have even been diagnosed with the following conditions by a physician: hypertension, cancer, diabetes, respiratory disease and cardiovascular disease. Functional limitations was classified based on information from 12 items that indicate having any difficulty doing specific activities by oneself and without any special equipment: (i) push or pull large objects, (ii) go out to things like shopping, (iii) participate in social activities, (iv) do things to relax at home or for leisure, (v) walk a quarter of a mile, (vi) walk up ten steps without resting, (vii) stand or be on feet for about two hours, (viii) sit for about two hours, (ix) stoop, bend, or kneel, (x) reach up over the head, (xi) use fingers to grasp or handle small objects, and (xii) lift or carry something as heavy as 10 pounds. If a person acknowledged having difficulty doing one or more of these activities was coded as “limited in any way” and considered to have functional limitations [16]. To prevent the exclusion of participants due to missing data (ranged 0% to 3.22%) a dummy category was created denoting lack of information for each variable.

### Statistical analyses

Analyses were conducted using STATA version 14.0 (Stata Corp, College Station, TX, USA) for Macintosh, with the level of statistical significance set at *p* < 0.05. Baseline characteristics of the study participants by disability in ADLs and IADLs were presented as mean (standard deviation) for continuous variables and as percentage for categorical variables.

Cox proportional regression models were used to estimate hazard ratios (HR) and their 95% confidence intervals (CI) for the association of each disability type (i.e., disability in ADLs and disability in IADLs) with mortality from all-cause, CVD, and cancer. Four models with sequential adjustment for potential confounders were fitted. Model 1 was adjusted for sociodemographic factors (sex, age, ethnicity, education and relationship status). Model 2 was additionally adjusted for lifestyle factors (smoking and alcohol consumption). Model 3 was additionally adjusted for health-related factors (BMI, hypertension, CVD, cancer, diabetes, and respiratory disease). And model 4 was additionally adjusted for functional limitations and for disability in ADLs or IADLs, as appropriate; that is, analyses for disability in ADLs were additionally adjusted for disability in IADLs (yes, no) and functional limitations (yes, no); and analyses for disability in IADLs were additionally adjusted for disability in ADLs (yes, no) and functional limitations (yes, no).

We also explored in detail the association between PA and with all-cause, CVD, and cancer mortality in people with and without disability in ADLs and IADLs. Firstly, we modelled restricted cubic spline Cox regressions, with knots at 10th, 50th, and 90th, to examine dose-response relationship between continuous values of PA and mortality for each subgroup (i.e., people with and without disability in ADLs and IADLs). Next, we examined the association between meeting PA recommendations and mortality, stratifying by disability; we run Cox regressions adjusting for the same models described above and considering as reference the participants who did not meet the recommended PA. Last, we examined whether PA attenuated the impact of disability on all-cause, CVD and cancer mortality risk. Thus, we tested the association of combined disability and PA recommendations with mortality risk, considering people without the specific disability and meeting PA recommendations as the reference group, as appropriate.

All analyses accounted for the complex survey design employed in NHIS by considering sample weights, and primary sampling units and stratum for variance estimation. Sample weights were first corrected by dividing by the number of pooled waves (i.e., twenty-two). To minimize reverse causation, sensitivity analyses were performed by excluding participants with a follow-up period < 2 years or removing from analyses people with CVD or cancer at baseline.

## Results

About 5.5% (*n* = 9,658) and 11.7% (*n* = 20,722) of participants had disability in ADLs and IALDs, respectively (Table 1). In brief, compared with participants without disability, those with disability were more likely to be women, older, of an ethnicity other than non-Hispanic white, with low educational level, not married, never smokers, and never or former drinkers; further, a higher percentage of people with disabilities had chronic diseases and functional limitations. As expected, a much lower compliance with the recommend PA was found among those with disability in ADLs or IADLs (all *p* < 0.05).

**Table 1:** Baseline characteristics of study participants and by disability types

	All	Disability in ADLs		Disability in IADLs	
		No	Yes	No	Yes
*n*	177,360	167,702	9,658	156,638	20,722
Women (%)	58.9	58.4	67.7	57.3	71.6
Age, years (mean ± SD)	71.3 ± 7.9	71.0 ± 7.7	76.3 ± 8.2	70.7 ± 7.6	76.2 ± 8.3
Ethnicity (%)					
Non-Hispanic white	74.3	74.9	62.5	75.2	67.8
Non-Hispanic black	12.2	11.8	19.0	11.6	17.1
Hispanic	9.4	9.2	13.4	9.2	10.9
Any other ethnic group	4.0	3.9	5.0	4.0	4.1
Educational level (%)					
Less than high school	23.6	22.8	38.3	21.7	38.0
High school or GED	29.9	30.0	27.8	30.1	28.2
More than high school	45.8	46.6	31.1	47.6	32.1
Relationship status (%)					
Married	44.5	45.4	28.4	47.5	22.2
Widowed/divorced/separated	48.8	47.9	64.1	46.0	70.2
Never married	6.5	6.5	7.1	6.4	7.4
Smoking (%)					
Never	50.9	50.8	52.6	50.7	52.4
Former	36.8	36.9	35.7	37.1	34.9
Currently	12.0	12.1	10.7	12.0	12.0
Alcohol drinking (%)					
Never	26.9	26.2	40.1	25.5	37.7
Former	24.6	23.9	36.5	23.2	35.0
Currently	47.3	48.8	21.2	50.2	25.5
BMI (%)^a^					
Normal weight	35.2	34.9	39.8	34.7	38.8
Overweight	36.1	36.6	26.1	37.2	27.4
Obesity	25.5	25.3	29.0	25.0	29.2
Chronic conditions					
Hypertension (%)	57.6	56.9	70.0	55.9	70.3
CVD (%)	30.9	29.4	57.3	30.9	53.8
Cancer (%)	19.7	19.5	23.8	19.2	23.7
Diabetes (%)	18.2	17.4	32.9	16.8	28.9
Respiratory disease (%)	16.6	16.1	25.2	15.4	26.1
Functional limitations (%)	62.2	60.3	95.2	57.9	95.4
PA, min/week (mean ± SD)	194.4 ± 344.6	203.4 ± 350.5	37.9 ± 147.6	213.5 ± 357.5	50.0 ± 164.3
Meeting PA recommendations (%)^b^	33.2	34.8	6.8	36.4	9.4

Abbreviations: *ADLs* activities of daily living, *IADLs* instrumental activities of daily living, *GED* general certificate of secondary education, *BMI* body mass index, *CVD* cardiovascular disease, *PA* physical activity

^a^Cut-points for overweight and obesity were 25 kg/m^2^ and 30 kg/m^2^, respectively

^b^% Meeting PA recommendations (≥ 150 min/week moderate-to-vigorous PA)

Over a mean (SD) follow-up of 8.02 (5.43) years, 66,694 deaths occurred from all-cause, 22,673 from CVD, and 13,845 from cancer. Having disability in ADLs or IADLs was associated with higher mortality, so that the increased risk of death ranged 47% to 49% for all-cause mortality, 33% to 41% for CVD, and 18% to 33% for cancer (Supplementary Table 1; model 4).

**Fig. 1:**
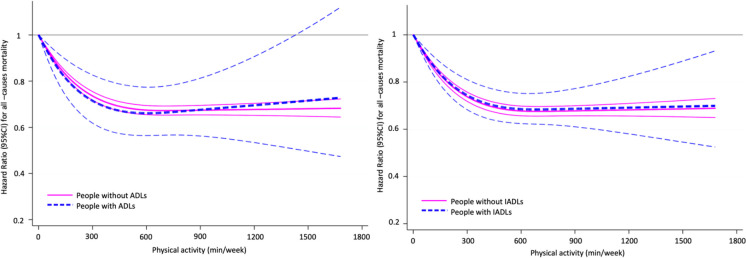
Dose-response associations between PA and all-cause mortality in people with and without ADLs, and IADLs. Thick lines show the hazard ratio values, ​​and thin lines the 95% confidence intervals. PA was truncated to 1,680 min/week. Analyses were adjusted for sex, age, ethnicity, education, marital status, smoking, alcohol consumption, body mass index, hypertension, cardiovascular diseases, cancer, diabetes, and any respiratory disease. Analyses for people with ADLs were additionally adjusted for IADLs (yes, no) and functional limitations (yes, no); analyses for people with IADLs were additionally adjusted for ADLs (yes, no) and functional limitations (yes, no). Abbreviations: ADLs, activities of daily livings; IADLs, instrumental activities of daily livings; PA, physical activity; CI, confidence interval

Dose-response associations between PA and all-cause mortality in people with and without disability in ADLs and IADLs are shown in Fig. 1. The shape of the dose–response associations between PA and all-cause mortality were inverse and not linear (all p for nonlinear trend < 0.001). In general, we observed a pronounced risk reduction up to 300–400 min/week of PA, and then a flattening of the trend with non-additional reduction at higher levels of PA in all analyses. Slight differences in the shape or the strength of the association were detected between those with and without disability in ADLs, but the curve was similar for people with and without disability in IADLs.

On other hand, dose-response associations of PA with CVD and cancer mortality showed some different patterns for people with and without disability in ADL and IADLs (Supplementary Figs. 2 and 3). For CVD mortality, in people with disability in ADLs or IADLs the risk decreases with a trend close to linearity, reaching greater reductions at high levels of PA than in people without disability in ADLs or IADLs. More inconsistent relationships were observed between the level of PA and cancer mortality in people with disability in ADLs or IADLs, such that the risk reduction becomes non-significant from values ​​around 800 min/week.

Associations between reaching PA recommendations and mortality, segmenting the analyses by disability are shown in Table 2. Among people with disability in ADLs, those meeting PA recommendations (vs not meeting) had 25%, 24% and 33% lower risk of all-cause, CVD, and cancer death, respectively. Values were 23%, 22% and 24% for those with disability in IADLs disability. Risk reductions associated with the recommended PA ranged 16% to 29% for people without the specific disability type. Results remained similar after excluding participants with a < 2-year follow-up (Supplementary Table 2), but some significant results dissipated after excluding those with CVD or cancer at baseline from analyzes (Supplementary Table 3). Maybe, due to loss of statistical power (i.e., low number of participants and deaths in some groups).

**Table 2:** Mortality risk reduction related to compliance with physical activity recommendations, according to the specific disability in older adults

	Not meeting PA recommendations		Meeting PA recommendations				
	n/deaths	HR (95%CI)	n/deaths	Model 1 HR (95%CI)	Model 2 HR (95%CI)	Model 3 HR (95%CI)	Model 4 HR (95%CI)
*All-cause mortality*							
Disability in ADLs							
No	109,392/45,736	1 (ref.)	58,310/14,701	**0.67 (0.66–0.69)**	**0.71 (0.69–0.72)**	**0.73 (0.71–0.74)**	**0.76 (0.74–0.78)**
Yes	9,003/5,922	1 (ref.)	655/335	**0.74 (0.65–0.84)**	**0.76 (0.66–0.86)**	**0.75 (0.66–0.85)**	**0.75 (0.66–0.86)**
Disability in IADLs							
No	99,617/39,519	1 (ref.)	57,021/14,054	**0.69 (0.67–0.70)**	**0.72 (0.71–0.74)**	**0.74 (0.72–0.75)**	**0.76 (0.74–0.78)**
Yes	18,778/12,139	1 (ref.)	1,944/982	**0.71 (0.66–0.77)**	**0.73 (0.68–0.79)**	**0.74 (0.69–0.80)**	**0.77 (0.71–0.83)**
*CVD mortality*							
Disability in ADLs							
No	109,392/15,865	1 (ref.)	58,310/4,615	**0.63 (0.60–0.65)**	**0.65 (0.63–0.68)**	**0.68 (0.66–0.71)**	**0.71 (0.69–0.75)**
Yes	9,003/2,078	1 (ref.)	655/115	**0.73 (0.57–0.93)**	**0.75 (0.59–0.96)**	**0.75 (0.59–0.95)**	**0.76 (0.60–0.97)**
Disability in IADLs							
No	99,617/13,580	1 (ref.)	57,021/4,381	**0.64 (0.61–0.67)**	**0.66 (0.64–0.69)**	**0.69 (0.66–0.72)**	**0.72 (0.69–0.75)**
Yes	18,778/4,363	1 (ref.)	1,944/349	**0.72 (0.63–0.82)**	**0.74 (0.65–0.84)**	**0.75 (0.66–0.86)**	**0.78 (0.68–0.89)**
*Cancer mortality*							
Disability in ADLs							
No	109,392/9,318	1 (ref.)	58,310/3,745	**0.78 (0.75–0.82)**	**0.83 (0.79–0.87)**	**0.82 (0.78–0.86)**	**0.84 (0.80–0.88)**
Yes	9,003/741	1 (ref.)	655/41	**0.67 (0.46–0.97)**	0.70 (0.48–1.01)	0.68 (0.46–1.01)	**0.67 (0.45–0.99)**
Disability in IADLs							
No	99,617/8,437	1 (ref.)	57,021/3,636	**0.79 (0.75–0.83)**	**0.84 (0.80–0.88)**	**0.83 (0.79–0.87)**	**0.84 (0.81–0.89)**
Yes	18,778/1,622	1 (ref.)	1,944/150	**0.74 (0.61–0.90)**	**0.77 (0.63–0.94)**	**0.75 (0.62–0.92)**	**0.76 (0.62–0.93)**

Abbreviations: *PA* physical activity, *HR* hazard ratio, *CI* confidence interval, *ADLs* instrumental activities of daily living, *IADLs* instrumental activities of daily living, *CVD* cardiovascular disease. Model 1 adjusted for sex, age, ethnicity, education, marital status; model 2 was adjusted as in model 1 plus smoking and alcohol consumption; model 3 was adjusted as in model 2 plus body mass index, hypertension, cancer, CVD, diabetes, and any respiratory disease; in model 4, analyses for people with ADLs were additionally adjusted for IADLs (yes, no) and functional limitations (yes, no), and analyses for people with IADLs were additionally adjusted for ADLs (yes, no) and functional limitations (yes, no). Statistically significant values are in bold (*p* < 0.05)

**Fig. 2:**
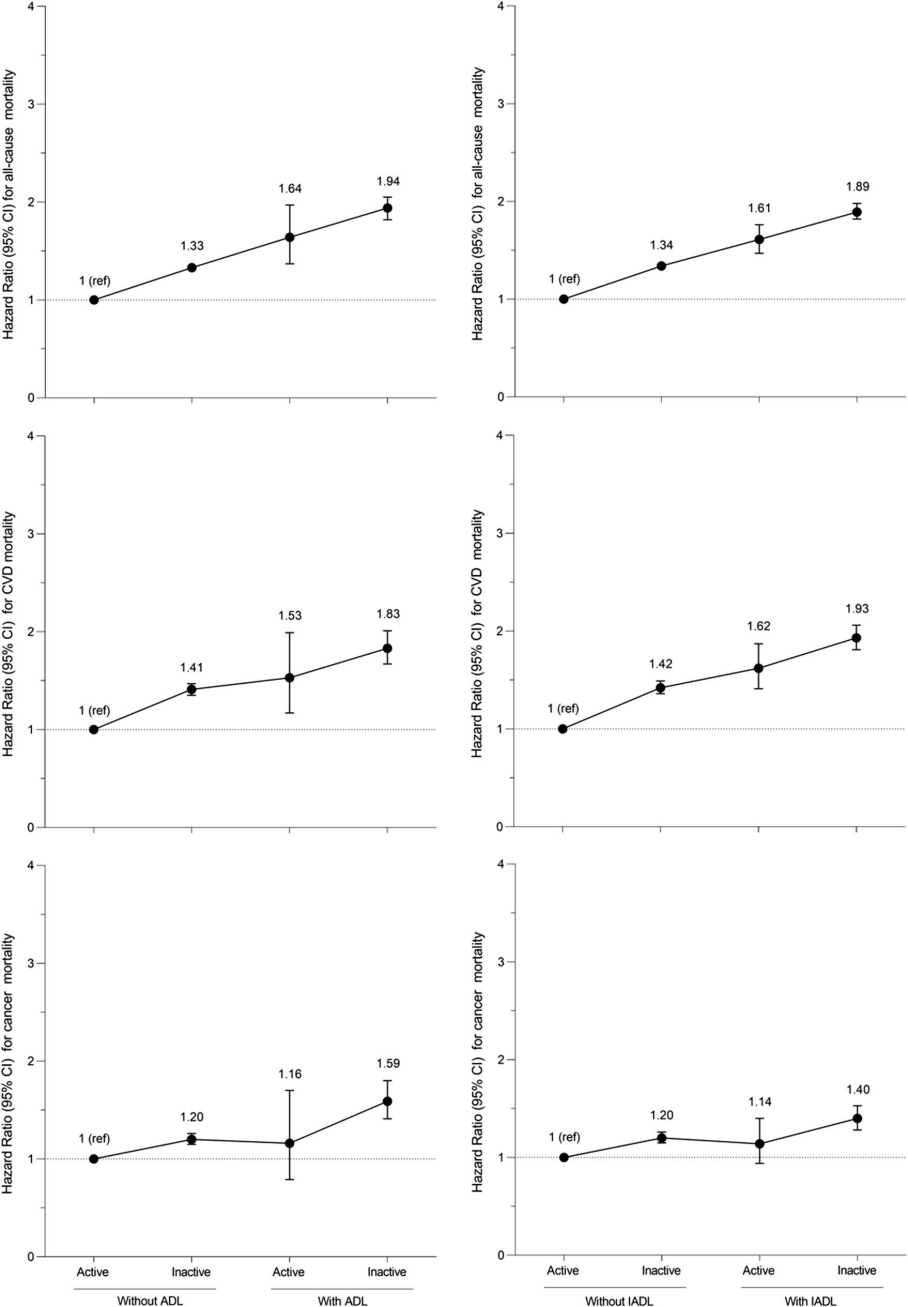
Mortality risk across combined physical activity recommendations and specific disability in older adults. Analyses were adjusted for sex, age, ethnicity, education, marital status, smoking, alcohol consumption, body mass index, hypertension, CVD, cancer, diabetes, and any respiratory. Analyses including people with ADLs were additionally adjusted for IADLs (yes, no) and functional limitations (yes, no), and analyses including people with IADLs were additionally adjusted for ADLs (yes, no) and functional limitations (yes, no). Abbreviations: PA, Physical Activity, CI, Confidence interval; ADLs, Activities of Daily Living; IADLs, Instrumental Activities of Daily Living; CVD, Cardiovascular Disease

Figure 2 shows the mortality risk associated with the combination of disability type and compliance with PA recommendations. Those with disability in ADLs or IADLs no meeting PA recommendations showed the highest all-cause mortality (HRs from 1.89 to 1.94), CVD (HRs from 1.83 to 1.93) and cancer (HRs from 1.40 to 1.59). However, those with disability who meet PA recommendations presented only moderately higher mortality than those without disability but not reaching PA recommendations. Sensitivity analyses excluding participants with a < 2-year follow-up (Supplementary Table 4), and those with CVD or cancer at baseline (Supplementary Table 5), yielded similar results, with some minor exceptions for cancer mortality.

## Discussion

In this analysis of a large US nationally representative study, we found a reduced mortality associated with meeting the recommended PA, which was similar in people with disability in ADLs or IADLs than in those without it. Interestingly, those older adults with disability who meet PA recommendations had only moderately higher mortality than those without disability who not achieved PA recommendations. These results emphasize the importance of compliance with PA recommendations to reduce the increased mortality risk of older people with disability in ADLs or IADLs.

PA has multiple positive effects on the ageing process and mortality [10, 21–24]. However, little is known about the association of PA with mortality in the older population with disability in ADLs or IADLs. A previous study in 3,752 noninstitutionalized individuals up to 60 years found a reduction in total and CVD death associated to PA among older adults with physical disability and highlighted that the beneficial impact of PA on mortality risk varies by disability type [9]. However, this study did not examine if the adherence to PA recommendations could attenuated mortality risk associated with disability in older adults and did not examine the dose-response relationship between PA and mortality. Our results showed that those with disability who meet PA recommendations present only a moderately higher mortality risk than those without disability but not meeting PA recommendations. Moreover, our results suggest that being physically active could be even more important for older adults with disability in ADLs and IADLs than those without disability, as meeting PA recommendations was related to 22% to 33% decreased mortality risk (all-cause, CVD or cancer) among people with disability, and to 16% to 29% among people without these disability types. These findings are extremely important because they provide the first evidence about the impact of meeting PA recommendations on mortality risk in older adults with disability in ADLs and IADLs.

We found a dose-response relationship between PA and mortality risk in older people with and without disability in ADLs and IADLs. The positive effect of PA on all-cause mortality started at low doses of PA in adults with and without disability in ADLs and IADLs. These results imply that even low levels of PA could reduce mortality risk in this population. We observed that high levels of PA (i.e., over 300–400 min/week) do not exert additional reductions in all-cause mortality in any sub-population. However, unlike people without disability in ADLs or IADLs, PA showed a close to a linear relationship with the risk of CVD death in older people with these disabilities, such that higher levels of PA caused greater reductions in CVD mortality risk. One potential reason why the association of PA on mortality risk varies by people with and without disability could be that PA has shown to have a greater impact on several chronic conditions (i.e., obesity, cognitive decline, frailty, etc.), which may occur more frequently in older adults with disability. Likewise, PA may be a stronger preventive factor in those with more severe disability [11, 12, 25]. Given the paucity of studies, further research is needed to study in depth the complex relationships between PA and mortality in older people with disability in ADLs and IADLs.

Our study also adds information on compliance with PA recommendations in US older adults with disability in ADLs and IADLs. In general, older adults with disability in ADLs and IADLs are even more physical inactive than older adults without these disability types, and rarely met the current PA guidelines [10, 26, 27]. For example, a US study found that only 2.5% of older adults with disability in ADLs meet the minimal amount of PA recommended [28], while other studies showed 15%, approximately, of older adults without disability met PA guidelines [29]. In our study, the prevalence of compliance with PA recommendations was much lower among older adults with disability in ADLs and IADLs than among those without disabilities. Specifically, only 6.8% and 9.4% of the older adults with disability in ADLs and IADLs meet the PA recommendations. Interventions to enhance PA in older adults with disability are needed, focusing on individual and interpersonal approaches through environmental and societal level interventions [30, 31]. Effective PA interventions for older adults with disability are crucial because they may increase their survival in addition to quality of life [13, 32].

This study has some strengths. First, we included a representative US sample of older adults with large follow-up; further, we considered a wide range of sociodemographic, behavioral, and health-related covariates in our analysis, and ran sensitive analyses aimed at reducing reverse causation. However, some limitations must be acknowledged, including the observational nature of the study which limits causal inference. PA was self-reported which could introduce accuracy and comprehension biases, leading to misclassification of PA levels; further, PA was only assessed at baseline, without considering possible changes in PA over time. Despite the extensive adjustment for covariates, some residual confounding could persist. Similarly, notwithstanding the sensitivity analyses performed, it is not possible to rule out some reverse causality in the results. Moreover, we included disabilities in ADLs and IADLs, but other specific limitations such as sensory impairment were not included. Of note is the absence of a measured of sedentary behavior. Sedentariness is associated with mortality, independent of PA levels [14, 33], so that future research should explore the combination of PA and sedentary time [34] to attenuate mortality risk associated with disability types in elderly.

In conclusion, in our large US population-based cohort, meeting PA recommendations mitigated the detrimental effects of disability in ADLs and IADLs on mortality in older adults. This finding adds to the existing limited evidence in mortality research on the importance of increasing PA and support the current recommendations that older adults with disability should be more active to enhance healthy aging longevity.

### Supplementary Information

Below is the link to the electronic supplementary material.

Supplementary file1 (DOCX 38 KB)

Supplementary file2 (DOCX 439 KB)

Supplementary file3 (DOCX 398 KB)

Supplementary file4 (DOCX 27 KB)

Supplementary file5 (DOCX 27 KB)

Supplementary file6 (DOCX 27 KB)

Supplementary file7 (DOCX 25 KB)

Supplementary file8 (DOCX 25 KB)

## Data Availability

The results of the present study are presented clearly, honestly, and without falsification, fabrication, or inappropriate manipulation. The findings and conclusions are those of the authors and do not necessarily represent the official position of the Centers for Disease Control and Prevention. The derived data generated in this research will be shared on reasonable request to the corresponding author. The public-use NHIS-National data (https://www.cdc.gov/nchs/nhis/) were accessed through the Integrated Public Use Microdata Series Health Surveys platform (https://nhis.ipums.org/nhis/).
