# Supplementary material for: The role of physical activity in the association between disability and mortality among US older adults: a nationwide prospective cohort study
Source: GeroScience. 2024 Jan 22;46(3):3275–85. doi: 10.1007/s11357-024-01072-9 (PMC11009203; doi:10.1007/s11357-024-01072-9)
Supplement: Supplementary file 1 — Supplementary file1 (DOCX 38 KB) [file 11357_2024_1072_MOESM1_ESM.docx]

**Supplementary figure 1.** Flowchart breaking down the United States National Health Interview Survey (NHIS) participants included in the present study. ^*^Including 8,234 participants with both disability in ADLs and IADLs. Abbreviations: ADLs, Activities of Daily Living; IADL, Instrumental Activities of Daily Living; NHIS, National Health Interview Survey; US, United States.

Total US NHIS sample (1997-2018)

(n=2,061,980)

(n=2,061,980)

Removed 1,872,371 participants

(not recorded, not selected as adult sample, or adults under 60 years)

Adults with 60 years old or older

(n=189,609)

Removed 6,005 participants without complete data on mortality

Participants eligible for mortality follow-up

(n=183,604)

Removed 785 participants without complete data on disability conditions

Analytical sample

(n=177,360)

With disability in ADLs

(n=9,658*)

With disability in IADLs

(n=20,722^*^)

Removed 5,459 participants without complete data on physical activity
