# Supplementary material for: The role of physical activity in the association between disability and mortality among US older adults: a nationwide prospective cohort study
Source: GeroScience. 2024 Jan 22;46(3):3275–85. doi: 10.1007/s11357-024-01072-9 (PMC11009203; doi:10.1007/s11357-024-01072-9)
Supplement: Supplementary file 3 — Supplementary file3 (DOCX 398 KB) [file 11357_2024_1072_MOESM3_ESM.docx]

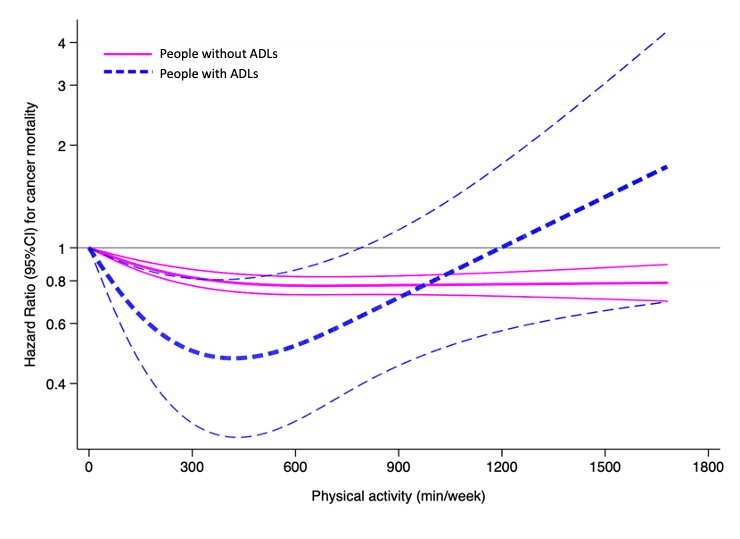

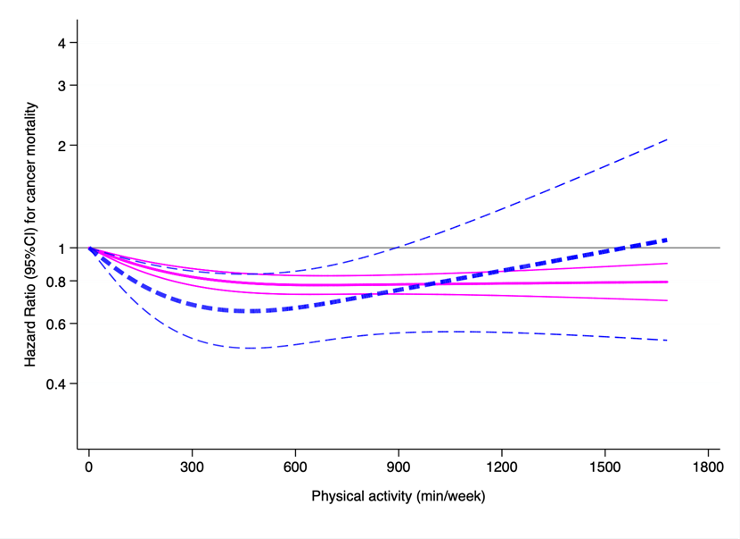


**Supplementary figure 3.** Dose-response associations between PA and cancer mortality in people with and without ADLs, and IADLs. Thick lines show the hazard ratio values, ​​and thin lines the 95% confidence intervals. PA was truncated to 1680 min/week. Analyses were adjusted for sex, age, ethnicity, education, marital status, smoking, alcohol consumption, body mass index, hypertension, cardiovascular diseases, diabetes, and any respiratory disease. Analyses for people with ADLs were additionally adjusted for IADLs (yes, no) and functional limitations (yes, no), and analyses for people with IADLs were additionally adjusted for ADLs (yes, no) and functional limitations (yes, no). Logarithmic scale was used for a better data values visualization. Abbreviations: ADLs, activities of daily livings; IADLs, instrumental activities of daily livings; PA, physical activity; CI, confidence interval.
