# Supplementary material for: The role of physical activity in the association between disability and mortality among US older adults: a nationwide prospective cohort study
Source: GeroScience. 2024 Jan 22;46(3):3275–85. doi: 10.1007/s11357-024-01072-9 (PMC11009203; doi:10.1007/s11357-024-01072-9)
Supplement: Supplementary file 4 — Supplementary file4 (DOCX 27 KB) [file 11357_2024_1072_MOESM4_ESM.docx]

**Supplementary table 1.** Mortality risk associated to specific disability type in older adults

|  | Disability in ADLs | |  | Disability in IADLs | |  |
| --- | --- | --- | --- | --- | --- | --- |
|  | No | Yes |  | No | Yes |  |
| *All-cause mortality* |  |  |  |  |  |  |
| n/deaths | 167,702/60,437 | 9,658/6,257 |  | 156,638/53,573 | 20,722/13,121 |  |
| Model 1, HR (95%CI) | 1 (ref.) | **2.45 (2.34-2.58)** |  | 1 (ref.) | **2.14 (2.07-2.21)** |  |
| Model 2, HR (95%CI) | 1 (ref.) | **2.35 (2.23-2.46)** |  | 1 (ref.) | **2.06 (1.99-2.12)** |  |
| Model 3, HR (95%CI) | 1 (ref.) | **2.03 (1.93-2.14)** |  | 1 (ref.) | **1.81 (1.75-1.87)** |  |
| Model 4, HR (95%CI) | 1 (ref.) | **1.49 (1.40-1.57)** |  | 1 (ref.) | **1.47 (1.41-1.52)** |  |
| *CVD mortality* |  |  |  |  |  |  |
| n/deaths | 167,702/20,480 | 9,658/2,193 |  | 156,638/17,961 | 20,722/4,712 |  |
| Model 1, HR (95%CI) | 1 (ref.) | **2.25 (2.10-2.41)** |  | 1 (ref.) | **2.06 (1.97-2.16)** |  |
| Model 2, HR (95%CI) | 1 (ref.) | **2.14 (2.00-2.30)** |  | 1 (ref.) | **1.98 (1.89-2.07)** |  |
| Model 3, HR (95%CI) | 1 (ref.) | **1.76 (1.64-1.89)** |  | 1 (ref.) | **1.66 (1.59-1.74)** |  |
| Model 4, HR (95%CI) | 1 (ref.) | **1.33 (1.23-1.44)** |  | 1 (ref.) | **1.41 (1.33-1.49)** |  |
| *Cancer mortality* |  |  |  |  |  |  |
| n/deaths | 167,702/13,063 | 9,658/782 |  | 156,638/12,073 | 20,722/1,772 |  |
| Model 1, HR (95%CI) | 1 (ref.) | **1.67 (1.52-1.84)** |  | 1 (ref.) | **1.48 (1.39-1.58)** |  |
| Model 2, HR (95%CI) | 1 (ref.) | **1.62 (1.48-1.78)** |  | 1 (ref.) | **1.43 (1.34-1.53)** |  |
| Model 3, HR (95%CI) | 1 (ref.) | **1.54 (1.40-1.70)** |  | 1 (ref.) | **1.36 (1.27-1.45)** |  |
| Model 4, HR (95%CI) | 1 (ref.) | **1.33 (1.19-1.49)** |  | 1 (ref.) | **1.18 (1.09-1.28)** |  |

Abbreviations: ADLs, activities of daily living; IADLs, Instrumental activities of daily living; HR, Hazard ratio; CI, Confidence interval, CVD, Cardiovascular disease. Model 1 was adjusted for sex, age, ethnicity, education, marital status; model 2 was adjusted as in model 1 plus smoking and alcohol consumption; model 3 was adjusted as in model 2 plus body mass index, hypertension, CVD, cancer, diabetes, and any respiratory disease; in model 4, analyses for people with ADLs were additionally adjusted for IADLs (yes, no) and functional limitations (yes, no); analyses for people with IADLs were additionally adjusted for ADLs (yes, no) and functional limitations (yes, no). Statistically significant values are in bold (p< 0.05).
