# Supplementary material for: The role of physical activity in the association between disability and mortality among US older adults: a nationwide prospective cohort study
Source: GeroScience. 2024 Jan 22;46(3):3275–85. doi: 10.1007/s11357-024-01072-9 (PMC11009203; doi:10.1007/s11357-024-01072-9)
Supplement: Supplementary file 6 — Supplementary file6 (DOCX 27 KB) [file 11357_2024_1072_MOESM6_ESM.docx]

**Supplementary table 3.** Mortality risk reduction related to compliance with physical activity recommendations, depending on the specific disability type in older adults, after removing people with CVD or cancer at baseline.

|  | Not meeting PA recommendations | | Meeting PA recommendations | | | |  |
| --- | --- | --- | --- | --- | --- | --- | --- |
|  | n/deaths | HR (95%CI) | n/deaths | Model 1  HR (95%CI) | Model 2  HR (95%CI) | Model 3  HR (95%CI) | Model 4  HR (95%CI) |
| *All-cause mortality* |  |  |  |  |  |  |  |
| Disability in ADLs |  |  |  |  |  |  |  |
| No | 61,390/21,649 | 1 (ref.) | 35,854/7,504 | **0.72 (0.69-0.74)** | **0.75 (0.72-0.77)** | **0.76 (0.73-0.78)** | **0.79 (0.76-0.81)** |
| Yes | 2,948/1,711 | 1 (ref.) | 248/106 | 0.81 (0.64-1.01) | 0.82 (0.65-1.04) | 0.80 (0.63-1.02) | 0.80 (0.63-1.03) |
| Disability in IADLs |  |  |  |  |  |  |  |
| No | 57,748/19,524 | 1 (ref.) | 35,327/7,258 | **0.72 (0.70-0.75)** | **0.75 (0.73-0.78)** | **0.76 (0.74-0.79)** | **0.78 (0.76-0.81)** |
| Yes | 6,590/3,836 | 1 (ref.) | 775/352 | **0.82 (0.72-0.93)** | **0.84 (0.74-0.96)** | **0.83 (0.73-0.95)** | **0.85 (0.75-0.98)** |
| *CVD mortality* |  |  |  |  |  |  |  |
| Disability in ADLs |  |  |  |  |  |  |  |
| No | 61,390/6,800 | 1 (ref.) | 35,854/2,157 | **0.67 (0.64-0.72)** | **0.70 (0.66-0.74)** | **0.71 (0.67-0.76)** | **0.74 (0.70-0.79)** |
| Yes | 2,948/510 | 1 (ref.) | 248/25 | 0.68 (0.41-1.12) | 0.68 (0.41-1.14) | 0.66 (0.40-1.10) | 0.66 (0.40-1.09) |
| Disability in IADLs |  |  |  |  |  |  |  |
| No | 57,748/6,118 | 1 (ref.) | 35,327/2,081 | **0.68 (0.64-0.73)** | **0.71 (0.66-0.75)** | **0.72 (0.68-0.77)** | **0.74 (0.69-0.79)** |
| Yes | 6,590/1,192 | 1 (ref.) | 775/101 | **0.77 (0.60-0.98)** | **0.78 (0.61-0.99)** | **0.77 (0.60-0.98)** | **0.78 (0.61-0.99)** |
| *Cancer mortality* |  |  |  |  |  |  |  |
| Disability in ADLs |  |  |  |  |  |  |  |
| No | 61,390/4,297 | 1 (ref.) | 35,854/1,891 | **0.83 (0.78-0.89)** | **0.88 (0.82-0.94)** | **0.88 (0.83-0.94)** | **0.89 (0.83-0.95)** |
| Yes | 2,948/163 | 1 (ref.) | 248/9 | 0.46 (0.20-1.04) | 0.46 (0.21-1.04) | 0.48 (0.21-1.08) | **0.42 (0.19-0.95)** |
| Disability in IADLs |  |  |  |  |  |  |  |
| No | 57,748/4,058 | 1 (ref.) | 35,327/1,847 | **0.83 (0.77-0.88)** | **0.87 (0.81-0.93)** | **0.87 (0.82-0.93)** | **0.88 (0.83-0.94)** |
| Yes | 6,590/402 | 1 (ref.) | 775/53 | 1.05 (0.76-1.47) | 1.07 (0.77-1.50) | 1.07 (0.77-1.50) | 1.02 (0.74-1.43) |

Abbreviations: PA, physical activity; HR, Hazard ratio; CI, Confidence interval; ADLs, Instrumental activities of daily living; IADLs, Instrumental activities of daily living; CVD, Cardiovascular disease. Model 1 adjusted for sex, age, ethnicity, education, marital status; model 2 was adjusted as in model 1 plus smoking and alcohol consumption; model 3 was adjusted as in model 2 plus body mass index, hypertension, diabetes, and respiratory disease; in model 4 analyses for people with ADLs were additionally adjusted for IADLs (yes, no) and functional limitations (yes, no), and analyses for people with IADLs were additionally adjusted for ADLs (yes, no) and functional limitations (yes, no). People with CVD (n=55,289) or cancer (n=21,631) at baseline were removed from the analyses. Statistically significant values are in bold (p< 0.05).
