# Supplementary material for: The role of physical activity in the association between disability and mortality among US older adults: a nationwide prospective cohort study
Source: GeroScience. 2024 Jan 22;46(3):3275–85. doi: 10.1007/s11357-024-01072-9 (PMC11009203; doi:10.1007/s11357-024-01072-9)
Supplement: Supplementary file 7 — Supplementary file7 (DOCX 25 KB) [file 11357_2024_1072_MOESM7_ESM.docx]

**Supplementary table 4**. Mortality risk associated to combined physical activity recommendations and specific disability type in older adults after removing people with a follow-up period lower than two years.

|  | No limitations/  Meet PA | | No Limitations/  Not meet PA | | Limitations/  Meet PA | | Limitations/  Not meet PA | |
| --- | --- | --- | --- | --- | --- | --- | --- | --- |
|  | n/deaths | HR (95%CI) | n/deaths | HR (95%CI) | n/deaths | HR (95%CI) | n/deaths | HR (95%CI) |
| *All-cause mortality* |  |  |  |  |  |  |  |  |
| Disability in ADLs | 53,280/13,471 | 1 (Ref.) | 98,300/39,449 | **1.27 (1.24-1.30)** | 499/231 | **1.41 (1.41-1.75)** | 6,450/3,761 | **1.56 (1.45-1.67)** |
| Disability in IADLs | 52,157/12,909 | 1 (Ref.) | 90,198/34,549 | **1.28 (1.25-1.31)** | 1,622/793 | **1.56 (1.41-1.73)** | 14,552/8,661 | **1.72 (1.65-1.81)** |
| *CVD mortality* |  |  |  |  |  |  |  |  |
| Disability in ADLs | 53,280/4,200 | 1 (Ref.) | 98,300/13,599 | **1.36 (1.31-1.43)** | 499/75 | 1.29 (0.94-1.76) | 6,450/1,379 | **1.58 (1.42-1.75)** |
| Disability in IADLs | 52,157/3,998 | 1 (Ref.) | 90,198/11,845 | **1.37 (1.32-1.44)** | 1,622/277 | **1.55 (1.33-1.82)** | 14,552/3,133 | **1.77 (1.65-1.90)** |
| *Cancer mortality* |  |  |  |  |  |  |  |  |
| Disability in ADLs | 53,280/3,318 | 1 (Ref.) | 98,300/7,602 | **1.12 (1.06-1.17)** | 499/25 | 0.95 (0.57-1.60) | 6,450/391 | **1.18 (1.00-1.39)** |
| Disability in IADLs | 52,157/3,230 | 1 (Ref.) | 90,198/6,988 | **1.12 (1.07-1.18)** | 1,622/113 | 1.05 (0.83-1.34) | 14,552/1,005 | **1.16 (1.04-1.29)** |

Abbreviations: PA, physical activity, CI, Confidence interval; ADLs, Instrumental activities of daily living; IADLs, Instrumental activities of daily living; CVD, Cardiovascular disease. Analyses were adjusted for sex, age, ethnicity, education, marital status, smoking status, alcohol consumption, body mass index status, hypertension, CVD, cancer, diabetes, and respiratory disease. Analyses for people with ADLs were additionally adjusted for IADLs (yes, no) and functional limitations (yes, no), and analyses for people with IADLs were additionally adjusted for ADLs (yes, no) and functional limitations (yes, no). People with a follow-up period lower than two years (n=18,831) were removed from the analyses. Statistically significant values are in bold (p< 0.05).
